# First detection of *Echinococcus multilocularis* in golden jackals (*Canis aureus*) in Bosnia and Herzegovina

**DOI:** 10.1016/j.ijppaw.2025.101099

**Published:** 2025-06-11

**Authors:** Naida Kapo, Jasmin Omeragić, Azra Bačić, Šejla Goletić, Adis Softić, Vedad Škapur, Toni Eterović, Jasna Salkić, Teufik Goletić

**Affiliations:** a University of Sarajevo – Veterinary Faculty, Sarajevo, Bosnia and Herzegovina; b Institute for Biomedical Diagnostics and Research – GENOM, Travnik, Bosnia and Herzegovina; c University of Sarajevo – Faculty of Agriculture and Food Science, Sarajevo, Bosnia and Herzegovina; d Faculty of Medicine, University of Tuzla, Department of Pathology, University Clinical Center, Tuzla, Bosnia and Herzegovina

**Keywords:** *Echinococcus multilocularis*, Golden jackal, PCR, NGS, Bosnia and Herzegovina, Expansion

## Abstract

*Echinococcus multilocularis*, the causative agent of alveolar echinococcosis (AE), is a parasitic zoonosis of increasing public health significance in Europe. Although previously confirmed in red foxes in Bosnia and Herzegovina, this study provides the first report of *E. multilocularis* in golden jackals (*Canis aureus*) in the country. Between December 2024 and March 2025, a total of 44 golden jackals were examined across 15 localities in Bosnia and Herzegovina, with *E. multilocularis* detected in 6.8 % of the samples from three sites in Western and Central Bosnia and Herzegovina. Adult *Echinococcus* spp. worms were first detected using the intestinal scraping technique and identified by microscopy. To confirm these findings and differentiate between *Echinococcus* species, DNA extracted from adult worms was then subjected to species-specific PCR targeting a fragment of the mitochondrial 12S ribosomal RNA gene. PCR-positive samples for *E. multilocularis* were further validated by next-generation sequencing (NGS) of a 203 bp amplicon of the 12S rRNA gene. These findings indicate an expanding distribution of *E. multilocularis* in Bosnia and Herzegovina, highlighting the role of golden jackals as definitive hosts for the parasite. Given the growing jackal population in the country and the increasing public health concerns, enhanced surveillance and further research are warranted, particularly regarding human cases of *E. multilocularis* infection, to better understand the associated epidemiological risks.

## Highlights

• First evidence of *Echinococcus multilocularis* in golden jackals from Bosnia and Herzegovina.
• Rising golden jackal population may increase *Echinococcus multilocularis* transmission.
• Expansion of *Echinococcus multilocularis* in golden jackals toward central Bosnia.

## Graphical abstract

**Figure:**
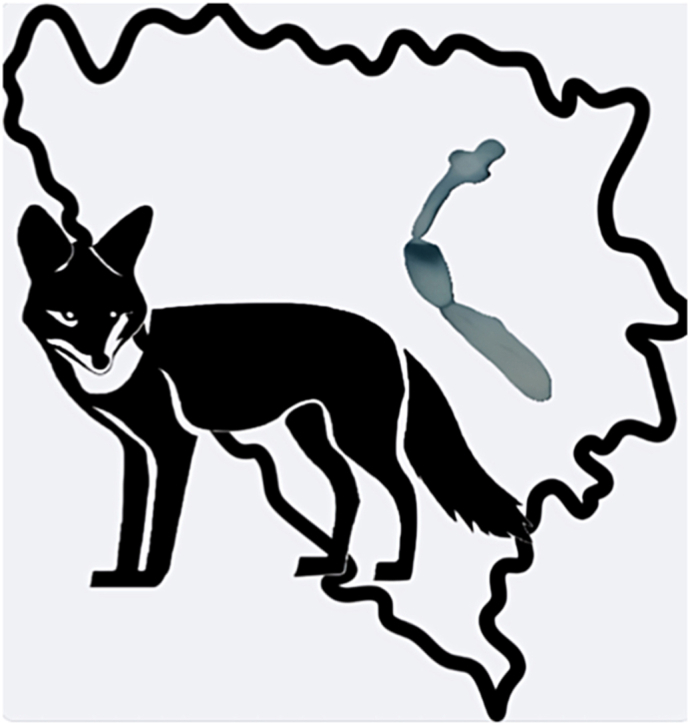

## 1 Introduction

*Echinococcus multilocularis* (Leuckart, 1863) is a cestode from the Taeniidae family responsible for alveolar echinococcosis (AE) in humans and other mammals (Bouwknegt et al., 2018). This disease, caused by the larval stage of *E. multilocularis*, is considered one of the most significant foodborne zoonoses in Europe and worldwide (Bouwknegt et al., 2018; Balog et al., 2021; van der Giessen et al., 2021). Humans are accidental hosts and develop AE after ingesting parasite eggs, either through direct contact with the definitive host or indirectly by consuming contaminated food or water (Torgerson et al., 2010).

In Europe, the red fox (*Vulpes vulpes*) plays a key role in the ecological cycle of *E. multilocularis*, while other canids, including the golden jackal (*Canis aureus*), the domestic dog (*Canis lupus familiaris*), the gray wolf (*Canis lupus*), and the raccoon dog (*Nyctereutes procyonoides*), act as secondary definitive hosts (Moloi et al., 2023). Earlier studies confirmed that golden jackals can serve as definitive hosts for this parasite, and more recent findings indicate their growing involvement in its transmission cycle (Széll et al., 2013; Oksanen et al., 2016; Balog et al., 2021; Caroline et al., 2022; Moloi et al., 2023).

In the Balkan region, the prevalence of *E. multilocularis* infection in red foxes and golden jackals shows similar values (Lalošević et al., 2016; Sindičić et al., 2018; Balog et al., 2021; Miljević et al., 2021). Studies conducted in Serbia have detected the presence of the parasite in approximately 14 % of the analyzed golden jackals, based on sample sizes of 28 and 64 animals (Lalošević et al., 2016; Miljević et al., 2021), while in Croatia, it was confirmed in 3.4 % of samples (Sindičić et al., 2018), indicating a relatively lower prevalence. In Slovenia, the most recent study reported 18 % positive cases in golden jackals, which aligns with the prevalence observed in the red fox population (Bandelj et al., 2022).

In recent years, cases of alveolar echinococcosis in humans have been reported in newly emerging endemic areas of the Balkan Peninsula, including Serbia, Croatia (Lalošević et al., 2016; Dušek et al., 2020; Balen et al., 2023; Lalošević et al., 2023). However, no human cases of *E. multilocularis* infection have been officially confirmed in Bosnia and Herzegovina. The lack of confirmed cases may be attributed to limited diagnostic methods, nonspecific clinical approaches, or underreporting, rather than a true absence of infections. Additionally, the lack of diagnostic data on the causative agents (i.e. *Echinococcus granulosus* sensu lato or *E. multilocularis*) in available medical records may contribute to diagnostic uncertainty. It should be noted that human cases of infection are typically confirmed only as 'echinococcosis' (without specifying the species), which further complicates species-level diagnosis (Omeragić et al., 2022; IPH FBiH, 2023/2024; IPH RS, 2022). In Bosnia and Herzegovina, *E. multilocularis* was first confirmed in red foxes in 2022 (Omeragić et al., 2022), but data on its presence in golden jackals remain unavailable. Given the significant increase in the golden jackal population in Bosnia and Herzegovina over the past decade (Kaczensky et al., 2024) and its growing role in parasite transmission (Széll et al., 2013; Oksanen et al., 2016; Caroline et al., 2022), there is a clear need to investigate its ecological impact. Since Bosnia and Herzegovina lacks an established surveillance and control system for this parasitic disease in animals, and human infection data remain limited, the aim of this study was to determine the presence of *E. multilocularis* in the golden jackal population. These findings could serve as an early indicator of a potential public health threat, considering the expansion of golden jackal habitats and population growth in Bosnia and Herzegovina.

## 2 Materials and methods

### 2.1 Samples

**Fig. 1:**
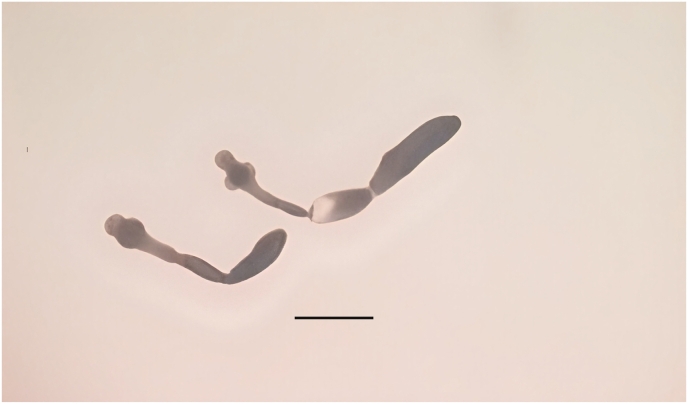
*Echinococcus multilocularis* adults isolated from golden jackals in Bosnia and Herzegovina. Scale bar: 0.5 mm.

Between December 2024 and March 2025, a total of 44 golden jackals were collected from 15 localities across Bosnia and Herzegovina (Fig. 1). The animals were hunted during the official pest control hunting season as part of the regular annual hunting harvest. The carcasses were then delivered to the Laboratory for Parasitology at the University of Sarajevo-Veterinary faculty, for further examination.

### 2.2 Parasitological methods

Following the autopsy, the intestines were removed and opened using gut scissors, and a visual examination was conducted to check for parasites. The small intestines of the golden jackals were divided into three portions of approximately equal length (anterior, middle, and posterior). The routine intestinal scraping technique (IST) was then performed, following the previously described protocol for detecting *E. multilocularis* adult worms in the small intestines (Tackmann et al., 2006). Briefly, the intestinal contents were carefully removed, and at least four samples per portion were collected at regular intervals of 5–10 cm, depending on the total length of the small intestine, by scraping the mucosa with a glass microscope slide. The material adhering to the slides was transferred to plastic Petri dishes and examined under a stereomicroscope (OLYMPUS, model CH20BIMF200) at a magnification of 40 to 100x. *Echinococcus* spp. tapeworms were extracted, rinsed with phosphate-buffered saline to eliminate any remaining gastrointestinal contents, and identified using morphological characteristics (Jones and Pybus, 2010) along with molecular analysis as detailed below.

### 2.3 Molecular methods

To confirm the morphological identification, PCR-based molecular techniques and NGS were employed. Parasitic DNA was extracted directly from three pools, each consisting of two adult parasites, using the QIAamp DNA Mini kit (Qiagen, Hilden, Germany), as described elsewhere (Beiromvand et al., 2011). The extracted DNA was stored at −20 °C until further analysis. Molecular detection and confirmation of the parasite were performed using PCR-based molecular methods adapted from Stieger et al. (2002), Stefanic et al. (2004) and Trachsel et al. (2007). All pools were analyzed using PCR assays that were either genus-specific (Stefanic et al., 2004) or species-specific (Stieger et al., 2002; Trachsel et al., 2007), allowing differentiation between *E. granulosus* and *E. multilocularis* by targeting the mitochondrial small subunit ribosomal RNA (12S rRNA) gene. All tested pools were positive exclusively for *E. multilocularis* (Table 1). Positive pools were subsequently prepared for sequencing and sequence assembly. Amplicons generated with *E. multilocularis*-specific primers EM-H15 and EM-H17 (Stieger et al., 2002) were purified and processed for sequencing using the PCR Barcoding Kit (Oxford Nanopore Technologies, Oxford, UK), following the manufacturer's protocol. Sequencing was conducted on the MinION Mk1C platform (Oxford Nanopore Technologies). Raw FAST5 data were basecalled, demultiplexed, and primers were trimmed using MinKNOW software (Oxford Nanopore Technologies). The 12S rRNA gene sequences were then assembled using CLC Genomics Workbench version 22.0.2 (Qiagen, Hilden, Germany), and the resulting consensus sequences were used for subsequent analyses.

**Table 1:** Species-specific PCR assays were employed to detect *E. multilocularis* and confirm the absence of *E. granulosus*.

Target species	PCR results	Gene	Primer designation	Reference
*E. multilocularis*	Positive (all pools)	12 S rRNA	EM-H15/EMH17	Stieger et al. (2002)
*E. granulosus*	Negative (all pools)	12 S rRNA	Cest4/Cest5	Trachsel et al. (2007)
*E. granulosus* (sheep strain)	Negative (all pools)	12 S rRNA	Eg1f/Eg1r	Stefanic et al. (2004)

## 3 Results

**Fig. 2:**
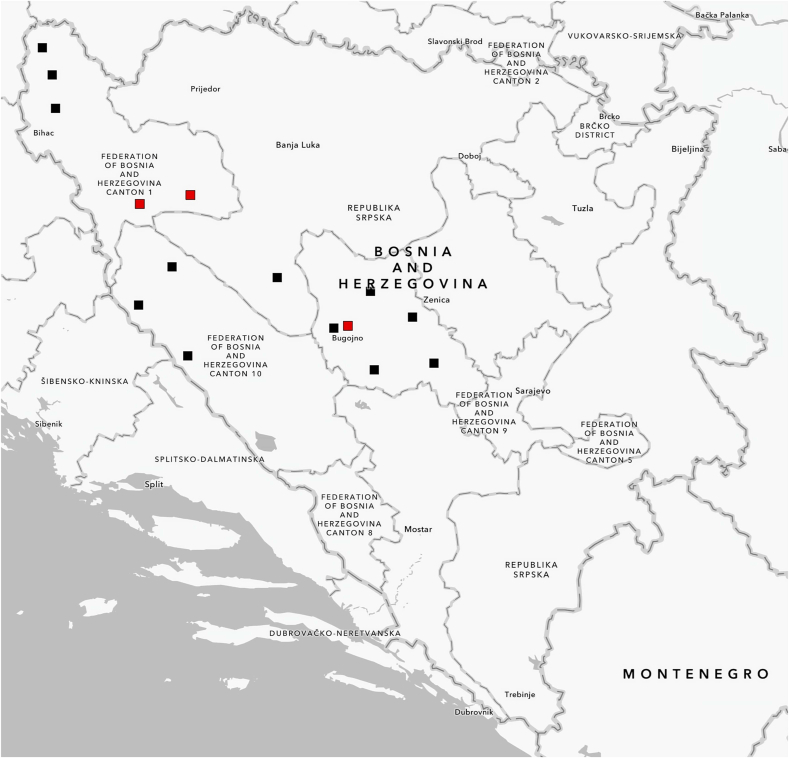
Map of Bosnia and Herzegovina with locations where *Echinococcus multilocularis* was confirmed in golden jackals (red squares). The study map was developed using ArcGIS® software (ESRI, Redlands, CA, USA), version 3.2.

Adult *E. multilocularis* parasites (Fig. 1) were identified in three golden jackals (3/44, 6.8 %) based on parasite size, scolex and strobila characteristics, and PCR-based molecular techniques. The number of adult *E. multilocularis* tapeworms found in these three jackals ranged from 15 to 163. The affected animals were found in the areas of Bugojno (Central Bosnia and Herzegovina), Bosanski Petrovac, and Sana (Western Bosnia and Herzegovina) (Fig. 2).

Three sequences,203 bp each, were assembled from three pooled samples and were compared using a BLAST search in the GenBank core nt database, confirming 100 % identity to each other and to other publicly available *E. multilocularis* 12S rRNA gene sequences in GenBank, including the one previously detected in a fox from Bihać (Northwestern Bosnia and Herzegovina) (Omeragić et al., 2022) (e.g., OP047920, LC720791.1, OP628495.1, OR911410.1, KY094610.1, OR911433.1, OR911404.1, OQ599952.1, MN444816.1). Since all sequences from all positive pools proved to be identical, only one was uploaded to GenBank (accesion no. PV588659).

## 4 Discussion and conclusions

Our study aimed to investigate the presence of *E. multilocularis* in the golden jackal population in Bosnia and Herzegovina, with a particular focus on regions with the largest golden jackal populations (Kaczensky et al., 2024), as well as areas where previous studies have confirmed the presence of this parasite in red foxes (Omeragić et al., 2022). The detection of *E. multilocularis* in golden jackals represents the first report of its presence in this species in Bosnia and Herzegovina, indicating the spread of the parasite toward the central parts of the country. These findings also suggest the local circulation of *E. multilocularis* among wild canids in Bosnia and Herzegovina.

The golden jackal's distribution in Europe has changed significantly in recent decades, with population numbers increasing in new areas. Historically native to southeastern Europe, including the Balkans, its population was reduced in the 1960s due to habitat loss and human activities (Spassov, 1989). However, in the past 30 years, factors such as climate change, land use changes, and increased food availability have led to a significant population growth (Spassov and Acosta-Pankov, 2019). This has resulted in migration toward Central, Western, and Northern Europe, where stable populations have formed. As the golden jackal population grows in Europe, its presence in Bosnia and Herzegovina is becoming increasingly dominant compared to the red fox. In Bosnia and Herzegovina, the golden jackal population has shown a growth trend in recent years, especially in the regions of Western and Central Bosnia (Kaczensky et al., 2024). Based on these data, golden jackals exhibit significant potential in the transmission of *E. multilocularis* in this area, given the high percentage of infected animals (6.8 %) compared to the previously observed rate in red foxes (1.75 %) (Omeragić et al., 2022). This suggests that golden jackals may become more significant than red foxes in spreading this pathogen in the Western and Central Bosnia regions of Bosnia and Herzegovina, where positive cases have been confirmed (Fig. 2). Reports from other countries provide data on the prevalence of *E. multilocularis* in golden jackals, with varying sample sizes. In Serbia, 4 out of 28 examined jackals were infected (14.3 %) (Lalošević et al., 2016), while in Slovenia, 7 out of 39 tested positive (18 %) (Bandelj et al., 2022). In Hungary, *E. multilocularis* was detected in 27 out of 173 examined jackals (15.6 %) (Balog et al., 2021), and in Croatia, 1 out of 29 tested positive (3.4 %) (Sindičić et al., 2018). These studies report a higher prevalence compared to our study. It's worth noting that the detection of *E. multilocularis* in Croatia was recorded near the border with Bosnia and Herzegovina, in the northwestern part of the country (Sindičić et al., 2018).

Our results, consistent with the aforementioned studies, highlight that golden jackals can successfully transmit *E. multilocularis* within and between habitats. Given the growing spread of the golden jackal population, the presence of this pathogen in golden jackals may have significant ecological and public health implications in Bosnia and Herzegovina, particularly in the context of the parasite's spread into new areas. Their role in maintaining *E. multilocularis* is particularly important, as they have been described as both intermediate and definitive hosts (Lalošević et al., 2016; Marinković et al., 2022).

In Bosnia and Herzegovina, infections with *Echinococcus* parasites in humans are insufficiently studied, and adequate data is lacking. Public health institutions in the country report only cases of echinococcosis in humans without specifying which *Echinococcus* species is involved. From 2022 to 2024, 16 cases of echinococcosis were confirmed, with the corresponding incidence of the disease ranging from 0.55 per 100,000 inhabitants during this period (IPH FBiH, 2023/2024; IPH RS, 2022). It is important to note that the highest number of human echinococcosis cases was reported in Western Bosnia in 2023, with an incidence of 0.8 to 0.55 per 100,000 inhabitants (IPH FBiH, 2023/2024). Given that we confirmed cases of *E. multilocularis* infection in both golden jackals and foxes in this region, it is crucial to determine whether these cases in humans are caused by hydatid or alveolar echinococcosis. The lack of species identification by healthcare professionals has led to insufficient education on the issue of *Echinococcus* infections. There is an assumption that the cases are linked to *E*. *granulosus* sensu lato, which often results in diagnostic confusion and the misunderstanding that there is only one species of *Echinococcus* involved.

In recent years, the confirmation of human cases of alveolar echinococcosis has become more prevalent in the region. In Serbia, the first case of echinococcosis was confirmed in a woman from the Sremska Mitrovica area, which is considered an endemic region for alveolar echinococcosis (Lalošević et al., 2023). Similarly, in Croatia, human cases of echinococcosis have been confirmed in a concentrated area in the rural eastern part of the Bjelovar-Bilogora County in central continental Croatia, which exceeds the highest reported regional incidence in Europe by more than double (Balen et al., 2023). This certainly indicates that alveolar echinococcosis has become an endemic disease in the continental parts of Croatia. Given that cases of alveolar echinococcosis in the region have mostly been reported in rural areas, where residents have been exposed to soil in these areas and vegetables grown in gardens near forests, and considering the lack of data in Bosnia and Herzegovina, it is crucial to take concrete steps to reduce the risk of infection. To address this, it is necessary to implement educational programs targeting both the general population and clinicians to raise awareness about the risks associated with this zoonosis. For example, educational campaigns could focus on safe practices when handling soil or gardening, as well as the importance of hand hygiene and cooking vegetables thoroughly. Additionally, the implementation of screening programs among the local population is recommended, with a particular focus on rural and isolated communities that are at greater risk of exposure to this parasite. These programs could involve periodic health check-ups and testing for *E. multilocularis* in at-risk populations. Furthermore, given the spread of this parasite, which poses a threat to public health, systematic monitoring of prevalence in both wildlife and domestic animals becomes of essential importance.

## CRediT authorship contribution statement

**Naida Kapo:** Writing – review & editing, Writing – original draft, Visualization, Validation, Supervision, Software, Methodology, Investigation, Formal analysis, Data curation, Conceptualization. **Jasmin Omeragić:** Writing – review & editing, Writing – original draft, Visualization, Validation, Supervision, Software, Resources, Project administration, Methodology, Investigation, Funding acquisition, Formal analysis, Data curation, Conceptualization. **Azra Bačić:** Writing – review & editing, Resources, Methodology, Investigation, Formal analysis, Conceptualization. **Šejla Goletić:** Writing – review & editing, Validation, Resources, Methodology, Investigation, Formal analysis, Conceptualization. **Adis Softić:** Writing – review & editing, Methodology, Investigation, Formal analysis. **Vedad Škapur:** Writing – review & editing, Methodology, Investigation, Formal analysis. **Toni Eterović:** Writing – review & editing, Methodology, Investigation, Formal analysis. **Jasna Salkić:** Methodology, Software, Validation, Writing – original draft, Writing – review & editing. **Teufik Goletić:** Writing – review & editing, Writing – original draft, Visualization, Validation, Supervision, Resources, Methodology, Investigation, Formal analysis, Data curation, Conceptualization.

## Ethics statement

This study was conducted under the Law on Animal Protection and Welfare of BH (“Official Gazette BH” issue number 316/09) and the Law on Hunting of the Federation of Bosnia and Herzegovina (“Official Gazette BH” issue number 4/06), and was approved by the Ethics Committee of the University of Sarajevo - Veterinary Faculty (Protocol Code 01-02-1242-3/23, January 01, 2024).

## Data availability statement

All datasets generated for this study are included in the article.

## Funding statement

The study was supported by the Grant of the 10.13039/100019950Environmental Protection Fund of the Federation of Bosnia and Herzegovina (Grant No. 01-09-2- 1581/2024) and the Ministry of Science, Higher Education, and Youth of the Sarajevo Canton (Grant No. 27-02-35- 35,137-11/22).

## Declaration of competing interest

The authors declare that they have no known competing financial interests or personal relationships that could have appeared to influence the work reported in this paper.
